# Polyphenols from Thinned Young Apples: HPLC-HRMS Profile and Evaluation of Their Anti-Oxidant and Anti-Inflammatory Activities by Proteomic Studies

**DOI:** 10.3390/antiox11081577

**Published:** 2022-08-15

**Authors:** Giulio Ferrario, Giovanna Baron, Francesca Gado, Larissa Della Vedova, Ezio Bombardelli, Marina Carini, Alfonsina D’Amato, Giancarlo Aldini, Alessandra Altomare

**Affiliations:** 1 Department of Pharmaceutical Sciences (DISFARM), Università degli Studi di Milano, Via Mangiagalli 25, 20133 Milan, Italy; 2 Plantex S.a.s., Galleria Unione 5, 20122 Milano, Italy

**Keywords:** thinned apples, polyphenols, anti-oxidant, anti-inflammatory, NRF2, NF-κB, proteomics

## Abstract

The qualitative profile of thinned apple polyphenols (TAP) fraction (≈24% of polyphenols) obtained by purification through absorbent resin was fully investigated by LC-HRMS in positive and negative ion mode and using ESI source. A total of 68 polyphenols were identified belonging to six different classes: flavanols, flavonols, dihydrochalchones, flavanones, flavones and organic and phenolic acids. The antioxidant and anti-inflammatory activities were then investigated in cell models with gene reporter for NRF2 and NF-κB and by quantitative proteomic (label-free and SILAC) approaches. TAP dose-dependently activated NRF2 and in the same concentration range (10–250 µg/mL) inhibited NF-κB nuclear translocation induced by TNF-α and IL-1α as pro-inflammatory promoters. Proteomic studies elucidated the molecular pathways evoked by TAP treatment: activation of the NRF2 signaling pathway, which in turn up-regulates protective oxidoreductases and their nucleophilic substrates such as GSH and NADPH, the latter resulting from the up-regulation of the pentose phosphate pathway. The increase in the enzymatic antioxidant cellular activity together with the up-regulation of the heme-oxygenase would explain the anti-inflammatory effect of TAP. The results suggest that thinned apples can be considered as a valuable source of apple polyphenols to be used in health care products to prevent/treat oxidative and inflammatory chronic conditions.

## 1. Introduction

Epidemiological studies indicate that consumption of apples and derivatives, such as apple juice, are beneficial in the treatment of some human diseases including CVD and related events, cancer and diabetes [1]. Beneficial effects have also been confirmed by intervention studies. Vallée Marcotte et al. [2] recently reviewed 20 intervention studies using apple juice, concluding that its consumption could exert some benefits on a variety of markers associated with the risk of developing chronic diseases including cardiovascular, cancer, and neurodegenerative diseases.

Recently, a regular consumption of 2–3 apples per day was associated to beneficial effects. In a randomized, controlled, crossover intervention study, in healthy subjects with mildly raised serum cholesterol concentrations, consumption of two Renetta Canada apples for eight weeks improved CVD risk factors, by reducing total and LDL cholesterol and ICAM-1 and increasing microvascular vasodilation [3]. Liddle et al. recently reported that consumption of three whole Gala apples per day for 6 weeks may be an effective strategy to mitigate inflammation in overweight and obese subjects, by reducing circulating biomarkers of inflammation and endotoxin exposure, including CRP, IL-6 and LBP and increasing the plasma antioxidant capacity [4].

Most of the documented beneficial effects of apples are attributed to the fraction of polyphenols represented by five main groups, namely, flavanols (catechins, epicatechin and procyanidins), flavonols (quercetin glycosides), phenolic acids (chlorogenic, gallic and coumaric acids), dihydrochalcones (phloretin glycosides) and anthocyanins (cyanidin) [5,6,7]. Apple polyphenols exert antioxidant, anti-inflammatory and lipid lowering effects as also confirmed by pre-clinical studies. In particular, isolated apple polyphenols (AP) have been proven to be effective in preventing/treating hypercholesterolemia [8,9], atherosclerosis in ApoE-deficient mice [10], colorectal cancer [11], non-alcoholic hepatitis [12], ulcerative colitis [13] and indomethacin-induced gastric damage [14].

At a molecular level, the antioxidant and anti-inflammatory activities of apple polyphenols can be partially explained by considering the role of polyphenols as activators of the NRF2 pathway [15]. NRF2 is a transcriptional factor associated with antioxidant enzymes playing a master role in redox homeostasis in cells. NRF2 and its principal negative regulator, KEAP1, play a central role in the maintenance of intracellular redox homeostasis and regulation of inflammation. NRF2 is proved to contribute to the regulation of the heme oxygenase-1 (HMOX1) axis, which is a potent anti-inflammatory target [16], and to inhibit oxidative stress by up-regulating antioxidant enzymes and co-factors [17]. Recently, there is an increase in the research literature regarding the regulation of NRF2 signaling pathways in different aspects of inflammation such as cytokines, chemokine releasing factors, MMPs and other inflammatory mediators affecting the NF-κB and MAPK networks to control inflammation, as recently reviewed by Saha et al. [18]. Activation of NRF2 by polyphenol compounds is mainly mediated by those compounds bearing an ortho-diphenol moiety which is oxidized to the corresponding quinone, which, being an electrophilic compound, reacts with the thiols of the KEAP1, thus releasing NRF2, which then translocates into the nucleus [19].

Hence, the phenolic enriched fraction from apples represents a valuable source of natural compounds with a beneficial effect against inflammation and oxidative stress, and they may be applied as a food supplement and/or functional ingredient for the treatment of chronic inflammatory diseases.

There is nowadays great interest in the bioactive compounds obtained from the waste products deriving from agriculture and the food industry (circular economy) [20]. Thinning young apples (around one month after blossom), which constitute a massive waste product of the apple production chain, is carried out in order to guarantee the output and to increase the quality of the harvested apples and are usually discarded in the orchard soil [21]. However, this has the negative effect of increasing the soil acidity and thus disturbing the microbial community, which in turn affects the growth of fruit trees [22]. Thinned young apples are particularly rich in polyphenols, more than 10-fold with respect to harvested apples. The total polyphenols and total antioxidant activity show a rapid decrease after 45 days from blossoming, stabilizing after 85 days, as found in the Fuji apples [23]. Recently, some papers have reported methods for purifying polyphenols from thinned apples by successive use of polyethylene and polyamide resins [24]. Moreover, studies have reported the beneficial effects of polyphenols from thinned apples which have a significant antibacterial activity and were found to be effective in inhibiting halitosis-related bacteria through damage to the cell membrane and hence should be a valuable waste material as a source of bioactive compounds [25]. Polyphenols from unripe apples were also found to exert anti-obesity activity in rats through the modulation of the fatty acid metabolism in the liver and the inhibition of the absorption of carbohydrates and fat [26].

Considering, on the one hand, the growing scientific interest in the apple and particularly in the apple polyphenol fraction as a source of bioactive compounds effective against inflammation and oxidative stress, and, on the other hand, taking into account that thinned apples are a waste product particularly rich in polyphenols, this work is aimed at fully characterizing the qualitative profile of polyphenols purified from thinned apples by a dual LC-HRMS approach (targeted and non-targeted), and at investigating their anti-inflammatory and antioxidant activities. Cell lines with gene reporters for NRF2 and NF-κB nuclear translocation were first used to assess the dose-dependent antioxidant and anti-inflammatory activities, and then to outline the molecular pathways involved by means of two integrated proteomic approaches, based on label-free and SILAC methodologies.

## 2. Material and Methods

### 2.1. Chemicals

Thinned Golden, Fuji, Bella del Bosco and Rosa Mantovana apples were sourced from farms located in Trentino-Alto Adige, Italy. Ultrapure water was prepared by a Milli-Q purification system (Millipore, Bedford, MA, USA). Protocatechuic acid, caffeic acid, *p*-coumaric acid, (+)-catechin, (−)-epicatechin, prunin (naringenin glucoside), phlorizin, phloretin, luteolin, cysteine (Cys), iodoacetamide (IAA), tris(2-carboxyethyl)phosphine (TCEP), tetraethylammonium bromide (TEAB), 3-(4,5-Dimethyl-2-thiazolyl)-2,5-diphenyl-2H-tetrazolium bromide (MTT), IL-1α, TNF-α, 6-hydroxy-2,5,7,8-tetramethyl-3,4-dihydrochromene-2-carboxylic acid (Trolox), ascorbic acid, catechin, sodium carbonate, Folin–Ciocalteu reagent, ferulic acid, naringenin-7-*O*-glucoside, quercetin, ethanol, methanol, phlorizin dihydrate, sodium acetate, acetic acid, 2,2-diphenyl-1-picrylhydrazyl (DPPH), CDDO-Me, rosiglitazone, formic acid (FA), trifluoroacetic acid (TFA), acetonitrile (ACN) and all ultra-pure-grade (99.5%) solvents used in LC-MS analysis were obtained from Merck KGaA, Darmstadt, Germany. Kaempferol, quercetin, quercetin-3-*O*-rhamnoside and quercetin-3-*O*-galactoside were purchased from Extrasynthese (Genay Cedex, France). S-TRAP™ columns were provided by Protifi (Huntington, NY, USA).

### 2.2. Isolation of Thinned Apple Polyphenols (TAP) Fraction

A total of 500 kg of thinned Golden, Fuji, Bella del Bosco and Rosa Mantovana apples were harvested 1 month after blossoming and stored at 2 °C for 1 month. The apples were thoroughly washed in a mixer with an aqueous solution containing 0.05% citric acid and then coarsely ground in a hammer mill. A 0.1% solution of pectinase was added to the resulting mush, which was then heated in a linear tunnel at 25 °C for 20 min.

The mass was then continuously forced through a filter with pressure of 200 bar and the squeezed juice was enzymatically treated with pectinase and centrifuged until a colorless solution was obtained. The liquid was then eluted through 20 L of XAD7 absorbent resin and washed with demineralized water until elimination of all the substances not retained by the resin. At the end of the elution with water, the retained substances (polyphenols) were eluted with 95% ethanol until all the bound components were recovered. Elution of polyphenols was checked by TLC. The hydro-alcoholic solution was concentrated under vacuum and then micronized.

### 2.3. Qualitative Analysis by LC-HRMS

The phytochemical profile of TAP fraction was performed by LC-HRMS as described by Baron et al. [27]. The extract was dissolved in methanol and diluted with mobile phase A to a final concentration of 2 mg/mL, added with Trolox (50 µM, final concentration) as internal standard (IS). The mixture was separated on a RP Agilent Zorbax SB-C18 column (150 × 2.1 mm i.d., 3.5 μm, CPS analitica, Milan, Italy) by an Ultimate 3000 system (Dionex, Sunnyvale, CA, USA) with a multistep program (80 min) of mobile phase A (H_2_O/HCOOH, 100/0.1, %*v/v*) and B (CH_3_CN/HCOOH, 100/0.1, %*v/v*). An LTQ Orbitrap XL mass spectrometer (Thermo Fisher Scientific, San Jose, CA, USA) was set to perform the analysis in data-dependent scan mode, and acquired the spectra in positive and negative ion mode. Full MS spectra were acquired by the FT analyzer (resolution 30,000 FWHM at *m/z* 400) in profile mode and in the range of *m/z* 120–1800. The MS/MS spectra of the 3 most intense ions exceeding 1 × 10^4^ counts of the full MS scan were acquired by the linear ion trap (LTQ) by using a normalized collision energy (CID) of 40 eV. A database containing the known components of apples and derivatives was built for the targeted data analysis [28,29,30,31,32,33,34,35,36,37,38,39,40,41,42,43,44]: the putative identification was obtained by comparing the accurate mass (5 ppm mass tolerance), the isotopic pattern and the fragmentation pattern with the compounds in the database. The identity of some molecules was confirmed by means of pure standards available in our laboratory. An untargeted data analysis was performed of the most intense ions not identified in the targeted analysis, as described by Baron et al. [27].

### 2.4. Quantitative Analysis of Total Polyphenol Content

#### 2.4.1. Colorimetric Analysis

The total polyphenol content was determined by the Folin–Ciocalteu colorimetric method, as reported by Baron et al. [45]; the calibration curve was built using catechin as a standard in a 1–1000 µg/mL range.

#### 2.4.2. HPLC Analysis

High-performance liquid chromatography (HPLC) coupled with a PDA detector was performed to evaluate the total polyphenol content. For the analysis, a methanolic solution of thinned apple polyphenols (TAP) fraction was diluted 1:4 in H_2_O/HCOOH, 100/0.1, %*v/v* (mobile phase A) to obtain a final concentration of 1 mg/mL.

The sample (injection volume, 10 µL) was analyzed in triplicate with a HPLC system (Surveyor, ThermoFinnigan Italy, Milan, Italy), equipped with a PDA detector (Surveyor, ThermoFinnigan Italy, Milan, Italy) and an RP Agilent Zorbax SB-C18 column (150 × 2.1 mm i.d., 3.5 μm, CPS analitica, Milan, Italy). The same gradient program described in Section 2.3 was used for the separation and quantification of TAP polyphenols, setting the PDA detector in a 200–600 nm range.

For the quantification, five calibration curves were built using a standard for each class of polyphenols characterizing the TAP extract: catechin for flavanols (10–100 µg/mL; λ_max_ 278 nm), ferulic acid for phenolic acids (1–10 µg/mL; λ_max_ 323 nm), naringenin-7-glucoside for flavanones (10–100 µg/mL; λ_max_ 283 nm), phlorizin for dihydrochalchones (10–100 µg/mL; λ_max_ 284 nm) and quercetin for flavonols (10–100 µg/mL; λ_max_ 371 nm).

Each chromatographic peak of the sample was assigned to the corresponding polyphenolic class on the basis of the UV spectrum. For the quantification, the AUC was interpolated using the calibration curve of the relative standard and the sum of the concentrations of all the compounds belonging to the same polyphenol class was calculated. The total polyphenol content was then expressed as a percentage (%), that is, mg of polyphenols present in 100 mg of extract.

### 2.5. Direct Radical Scavenging Activity

The antioxidant activity was evaluated with the DPPH assay. A solution of thinned apple polyphenols (TAP) fraction was prepared with H_2_O:EtOH 50:50 (%*v/v*) to obtain final concentrations in the range 1–20 µg/mL. An aliquot of TAP extract solution (500 µL) was mixed with 1 mL of acetate buffer (pH 5.5, 100 mM) and 1 mL of EtOH. Then, 500 µL of DPPH (500 µM, ethanolic solution) was added and samples were kept in the dark for 90 min. A Shimadzu UV 1900 spectrophotometer (Shimadzu, Milan, Italy) was used for reading the absorbance at 517 nm. Trolox and ascorbic acid were used as reference antioxidant compounds. The percentage of inhibition (I%) was calculated as expressed by Equation (1) and the results are reported as mean ± SD.

(1) $$I\%=\frac{Abs\;\left(blank\;sample\right)-Abs\;\left(sample\right)}{Abs\;\left(blank\;sample\right)}\times 100$$

### 2.6. Cell-Based Assays

#### 2.6.1. MTT Assay

The effect of thinned apple polyphenols (TAP) fraction on the cell viability for all the concentrations tested in the anti-inflammatory and antioxidant assays was verified by MTT assay on R3/1-NF-κB cells and HEK293 cells in transparent 96-well plates seeded with 4000 cells/well and 10,000 cells/well, respectively. Cells were treated with different concentration of the extract (1 µg/mL–250 µg/mL) for 18 h in complete medium (DMEM 10% FBS, 1% penicillin/streptomycin). Subsequently, media were removed and only for R3/1-NF-κB cell line, one wash with 100 μL PBS occurred. Then, 100 µL of DMEM, not supplemented with FBS and penicillin/streptomycin, was added to each well and the 4 h incubation started after the addition of 11 µL, 5 mg/mL MTT reagent. After medium removal, cells were lysed using 100 µL of a solution composed of DMSO, 8 mM HCl and 5% TWEEN20. The 96-well plate was shaken for 15 min in a plate shaker in the dark and the absorbance at 575 nm and 630 was measured using a plate reader (BioTek’s PowerWave HT, Winooski, VT, USA). Cells incubated with DMSO (0.1%) were used as a control for 100% cell proliferation, while cells incubated with DMSO (3%) were used as a negative control.

#### 2.6.2. NRF2 Gene Reporter Cell Model

Thinned apple polyphenols (TAP) extract was evaluated for its ability to modulate the antioxidant response mediated by NRF2 activation using NRF2/ARE Responsive Luciferase Reporter HEK293 stable cell line (Signosis, Santa Clara, CA, USA) as previously described [46]. Briefly, HEK293 cells (10,000 cells/well) were treated with the extract (concentrations between 1 and 250 µg/mL). CDDO-Me 75 (bardoxolone methyl) 75 nM was used as a positive control [47]. After adding ONE-Glo™ Luciferase Assay Substrate (purchased from Promega Corporation, Madison, WI, USA) (100 µL/well), luciferase measurement was performed with a luminometer (Wallac Victor2 1420, Perkin-Elmer™ Life Science, Monza, Italy). Experiments were carried out with biological and technical replicates; values are reported as mean ± SD compared to untreated control cells. One-way ANOVA with Bonferroni’s multiple comparisons test (*p* < 0.05 was considered significant) was used for the statistical analysis.

#### 2.6.3. NF-κB Gene Reporter Cell Model

The in vitro anti-inflammatory activity of the TAP extract was evaluated by using a cell model previously described [27]. Briefly, R3/1 NF-κB cells (5000 cells/well) were pre-treated for 18 h with different concentrations of the extract (1–250 µg/mL) in complete medium (DMEM 10% FBS, 1% l-glutamine, 1% Penicillin/Streptomycin). Rosiglitazone 10 µM was used as a positive control [27]. Then, cells were stimulated for 6 h with 10 ng/mL IL-1α, and for 6 and 24 h with 10 ng/mL TNF-α. Luciferase measurements was performed with a luminometer (Wallac Victor2 1420, Perkin-Elmer™ Life Science, Monza, Italy) after adding 100 µL of ONE-Glo™ Luciferase Assay Substrate (purchased from Promega Corporation, Madison, WI, USA). Experiments were carried out with biological and technical replicates; values are reported as mean ± SD compared to untreated control cells. One-way ANOVA with Bonferroni’s multiple comparisons test (*p* < 0.05 was considered significant) was used for the statistical analysis.

### 2.7. Quantitative Proteomic Studies

#### 2.7.1. SILAC Culture

The R3/1 NF-κB reporter cell line used for SILAC experiments was cultured in DMEM for SILAC supplemented with 10% FBS, 1% pen/strep and 1% sodium pyruvate; the medium was completed by adding 0.5 mL of heavy or light l-lysine and l-arginine 1000X stock solution diluted in PBS. The final concentration of the light amino acids was 84 mg/mL for arginine and 146 mg/mL for lysine. For the heavy condition, both amino acids (^13^C_6_ ^15^N_2_ lysine and ^13^C_6_ ^15^N_4_ arginine) were diluted in the medium up to the working concentration of 88 mg/mL and 151.3 mg/mL for the heavy arginine and lysine, respectively; this difference in concentration is due to the different molecular weights of the amino acids [48,49]. The cell line was cultivated for at least 9 passages and the incorporation rate was checked: more than 95% of all peptides are required to be labeled.

##### SILAC Experimental Design

**Figure 1:**
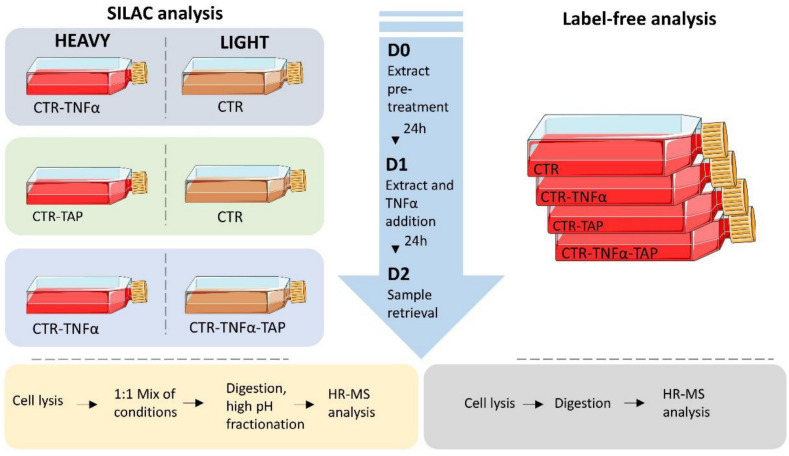
Experimental design of the quantitative proteomic analysis based on the two orthogonal approaches applied, namely, SILAC (**left side**) and LFQ (**right side**), interspersed by a timeline describing the three basic treatment steps. Below is a schematic of the sample processing protocol.

Each condition tested was cultivated in biological triplicate, and once 70% confluence was reached in a T75 flask, the cells were treated according to the planned experimental design, as shown in Figure 1. Overall, the conditions chosen were as follows: (i) *Control (CTR)*, i.e., untreated cells; (ii) *Thinned apple polyphenols (TAP) extract treatment (CTR-TAP),* i.e., cells that underwent a double 24 h treatment with the extract at a 200 µg/mL concentration; (iii) *Inflammation* (CTR-TNF), condition achieved treating cells for 24 h with TNF-α 0.01 µg/mL; (iv) *Thinned apple polyphenols (TAP) extract treatment of inflamed cells (CTR-TNF-TAP)*, consisting of the 24 h pre-treatment with the extract at 200 µg/mL and then with TNF-α at 0.01 µg/mL for another 24 h.

The metabolic labeling strategy was planned so that all conditions are compared with each other in order to have a comprehensive and meaningful pathway modulation overview (Figure 1). All the experiments were planned to finish in the same day.

After light or heavy amino acid incorporation, the flasks were washed twice with warmed PBS and the cell was detached using 2 mL of warmed trypsin incubated at 37 °C for 5 min. Cells were then recovered. After diluting the trypsin with 8 mL of complete medium, the suspension was centrifuged for 5 min at 300× *g* at room temperature and the pellet was then washed 5 times with 5 mL of 4 °C PBS. The cell pellets were then lysed using a suitable S-trap (Protifi) buffer composed of SDS 5%, TEAB 50 mM, MgCl 2 mM, one complete™ EDTA-free protease inhibitor cocktail tablet (Sigma-Aldrich, Milan, Italy) and 100 Unit of benzonase to reduce nucleic acids content that could occlude the S-trap column. The lysates completely solubilized after 30 min of mechanic resuspension in a tube rotator at 4 °C and were centrifuged at 10,000× *g* for 15 min at 4 °C in a refrigerated centrifuge. Once the lysates were ready, the protein concentration of differentially labeled samples was assessed using the BCA kit and the conditions were mixed in a 1:1 ratio to obtain 100 µg of proteins before any further processing.

Each mixture was prepared by mixing one specific biological replicate with another at random.

##### S-Trap Digestion

Samples collected from all the prepared incubation mixtures were then processed according to the bottom-up proteomics procedure. Sample preparation begins with the solubilization of samples in 5% SDS followed by further denaturation by acidification and subsequent exposure to a high concentration of methanol. The collected incubation mixtures were then solubilized 1:1 in lysis buffer (10% SDS, 100 mM TEAB). The reduction of disulfide bridges was performed by adding 5 µL of the reducing solution of tris(2-carboxyethyl)phosphine (c.f. 5 mM TCEP in 50 mM AMBIC) and incubating the mixtures under slow shaking in a Thermomixer for 10 min at 95 °C. Next, a volume of 5 μL of iodoacetamide solution (c.f. 20 mM IAA in 50 mM AMBIC) was added, with the aim of alkylating the free thiol residues; incubation in this case was carried out for 45 min at room temperature in the dark. Proteins were further denatured by acidification to pH < 1 by adding a 12% phosphoric acid solution in water (1:10 relative to sample volume). The next step consisted of the sample loading: 165 µL of the binding buffer (90% methanol, 10% TEAB 1 M) and 25 µL of sample were simultaneously added onto the spin columns, then centrifuged at a speed of 4000× *g* for 1 min at 15 °C; this step was repeated until the protein sample was fully loaded onto the columns.

After that, three washing steps by adding 150 µL of binding buffer onto the S-TRAP columns, followed by centrifugation (1 min, 4000× *g*, 15 °C), were performed to remove all the excess of unbound sample. At this point, the proteolytic digestion step started by adding a volume of 25 µL containing 1 µg of trypsin (sequencing-grade trypsin, Roche) diluted in 50 mM AMBIC. Upon addition of the protease, the physical confinement within the submicron pores of the trap forces substrate and protease interaction to yield rapid digestion; therefore, protein digestion requires much shorter incubation times, i.e., 1.5 h at 47 °C under slow stirring (400 rpm). The peptide mixture was recovered by loading two different solutions—40 µL of elution buffer 1 (10% H_2_O, 90% ACN, 0.2% FA) and 35 µL of elution buffer 1 (60% H_2_O, 40% ACN, 0.2% FA)—onto the columns (elution and 1′ centrifugation, 4000× *g*, 15 °C). The collected peptide mixtures were dried in the SpeedVac (Martin Christ., Osterode am Harz, Germany) at 37 °C and stored at −80 °C until analysis.

##### High pH Fractionation

The digested peptide mixtures obtained by means of the S-Trap™ micro-spin column digestion strategy were fractionated using the Pierce™ High pH Reversed-Phase Peptide Fractionation Kit according to the manufacturer’s instructions, with one additional fraction at 80% ACN.

##### nLC-HRMS Orbitrap Elite™ Mass Spectrometer Analysis

Tryptic peptides, resuspended in an appropriate volume (18 µL, sufficient for two technical replicates) of 0.1% TFA mobile phase, were analyzed using a Dionex Ultimate 3000 nano-LC system (Sunnyvale, CA, USA) connected to the Orbitrap Elite™ Mass Spectrometer (Thermo Scientific, Brema, Germania) equipped with an ionization source, the nanospray (Thermo Scientific Inc., Milano, Italia).

For each sample, 5 µL of solubilized peptides was injected in triplicate onto the Acclaim PepMap™ C18 column (75 µm × 25 cm, 100 Å pores, Thermo Scientific, Waltham, MA, USA), “protected” by a pre-column, the Acclaim PepMap™ (100 µm × 2 cm, 100 Å pores, Thermo Scientific, Waltham, MA, USA), thermostatically controlled at 40 °C. The chromatographic method used a binary pump system (LC/NC pumps) and started from sample loading onto the pre-column (3 min) using the loading pump with a flow rate of 5 µL/min of mobile phase consisting of 99% buffer A_LC, 0.1% TFA/1% buffer B_LC and 0.1% FA in ACN. After the loading valve switching, peptide separation was performed by the Nano Column Pump (NC_pump) with a 117 min linear gradient (0.3 µL/min) of buffer B_NC_pump (0.1% FA in ACN) from 1% to 40%, and a further 8 min of linear gradient from 40% to 95% (Buffer B_NC_pump). Then, 5 min at 95% of buffer B_NC_pump to rinse the column followed the separative gradient, and the last 7 min served to re-equilibrate the column to initial conditions. The total run time was 144 min. A washout injection with pure acetonitrile (5 µL) was performed between sample injections.

The nanospray ionization source was set as follows: positive ion mode, spray voltage at 1.7 kV; capillary temperature at 220 °C, capillary voltage at 35 V; tube lens offset at 120 V. The orbitrap mass spectrometer operating in data-dependent acquisition (DDA) mode was set to acquire full MS spectra in “profile” mode over a scan range of 250–1500 *m/z*, with the AGC target at 5 × 10^5^, and resolution power at 120,000 (FWHM at 400 *m/z*). Tandem mass spectra were instead acquired by the linear ion trap (LTQ), set to automatically fragment in CID mode the ten most intense ions for each full MS spectra (over 1 × 10^4^ counts) under the following conditions: centroid mode, normal mode, isolation width of the precursor ion of 2.5 *m/z*, AGC target 1 × 10^4^ and normalized collision energy of 35 eV. Dynamic exclusion was enabled (exclusion dynamics for 45 s for those ions observed 2 times in 10 s). Charge state screening and monoisotopic precursor selection were enabled, and singly charged and unassigned charged ions were not fragmented. Xcalibur software (version 3.0.63, Thermo Scientific Inc., Milan, Italy) was used to control the mass spectrometer.

#### 2.7.2. LFQ Analysis

For the LFQ (Label-Free Quantitative Proteomics) experiment, the same cell line (R3/1 NF-κB reporter cell line) was cultivated in biological triplicate in T-25 flasks and the same experimental conditions were tested for SILAC ((i) *Control, CTR*; (ii) *Thinned apple extract treatment (CTR-TAP)*; (iii) *Inflammation (CTR-TNF)*; (iv) *Thinned apple extract treatment of inflamed cells (CTR-TNF-TAP),* as shown in Figure 1). Except for the labeling strategy, the lysate preparation procedure is the same, as is the proteolytic digestion performed exploiting the potential of the S-Trap™ Micro Spin Column Digestion Protocol.

##### nLC-HRMS Orbitrap Fusion™ Tribrid™ Mass Spectrometer Analysis

Tryptic peptides were analyzed using a Dionex Ultimate 3000 nano-LC system (Sunnyvale, CA, USA) connected to an Orbitrap Fusion Tribrid Mass Spectrometer (Thermo Scientific, Bremen, Germany) equipped with a nano-electrospray ion source according to the procedure previously described [50].

#### 2.7.3. Data Analysis

For both the SILAC and label-free analysis, the instrumental raw files were processed by MaxQuant software v.1.6.6.0 set on *Rattus_Norvegicus* database (Uniprot taxonomy ID: 10116) against the Andromeda search engine. Protein quantification using SILAC (3 biological × 2 technical replicates for each condition) was based on the ratio of peptides’ peak intensities in the mass spectrum reflecting the relative protein abundance, while for the label-free approach (3 biological × 3 technical replicates for each condition), the quantification was based on LFQ intensity. In both analyses, trypsin was specified as proteolytic enzyme, cleaving after lysine and arginine except when followed by proline, and up to two missed cleavages were allowed along with match between run option. The precursor ion tolerance was set to 5 ppm while the fragment tolerance was set to 0.5 Da. Carbamidomethylation of cysteine was defined as fixed modification, while oxidation of methionine and acetylation at the protein N-terminus were specified as variable modifications. For the SILAC experiment, only the multiplicity of the labels was set to 2, and Arg10 and Lys8 were selected as heavy aminoacidic residues. Interpretation of the results was performed using Perseus (v.1.6.1.43, Max Plank Institute of Biochemistry, Martinsried, Germany). For the SILAC analysis, the normalized ratio count was selected, transformed in log2, filtered for minimum of 3 valid values and then a one sample *t*-test was performed with Benjamini–Hochberg FDR for truncation with a threshold of 0.05. LFQ analysis was validated by applying a two-sample t-test of the log2 LFQ intensities. The network protein analyses related to significantly altered proteins were carried out by means of Cytoscape v.3.9.1 and the ingenuity pathways analysis (IPA) (QIAGEN Aarhus Prismet, Aarhus, Denmark, September 2021) licensed software based on the Gene Ontology database. All statistical analyses of the inflammatory assays were conducted using GraphPad prism 8. The Venn diagrams with proportional areas were obtained using the bioVenn package for R [51].

##### Cytoscape Analysis

The protein–protein interaction network of the up-regulated proteins (log2 *Fold-Change* > 0.5) and down-regulated proteins (log2 *Fold-Change* < −0.5) was obtained by importing a list of Protein IDs into Cytoscape v.3.9.1 (http://cytoscape.org, accessed on 15 February 2022). We used the embedded STRING interaction database (http://apps.cytoscape.org/apps/stringApp, accessed on 15 February 2022) with a default confidence cut-off score of 0.4. *Rattus Norvegicus* was selected as a reference database with GO, KEGG, PFAM, WikiPathways, Reactome and InterPro cluster terms selected as functional annotations for the enrichment analyses.

##### Ingenuity Pathways Analysis (IPA)

The core analyses performed by IPA, using the differentially expressed proteins in the uploaded dataset, assess signaling pathways, molecular interaction network and biological functions that can likely be perturbed. The overall activation/inhibition states of canonical pathways are predicted through a z-score algorithm. This z-score is used to statistically compare the uploaded dataset with the pathway patterns. The pathways are colored to indicate their activation z-scores: orange predicts a gain of function, and blue a loss of function. The pathway is activated when molecules’ causal relationships with each other (i.e., activation edge and the inhibition edge between the molecules based on literature findings) generate an activity pattern for the molecules and the end-point functions in the pathway.

## 3. Results and Discussion

### 3.1. Qualitative Profile of Polyphenols Determined by Targeted LC-HRMS Analysis

**Figure 2:**
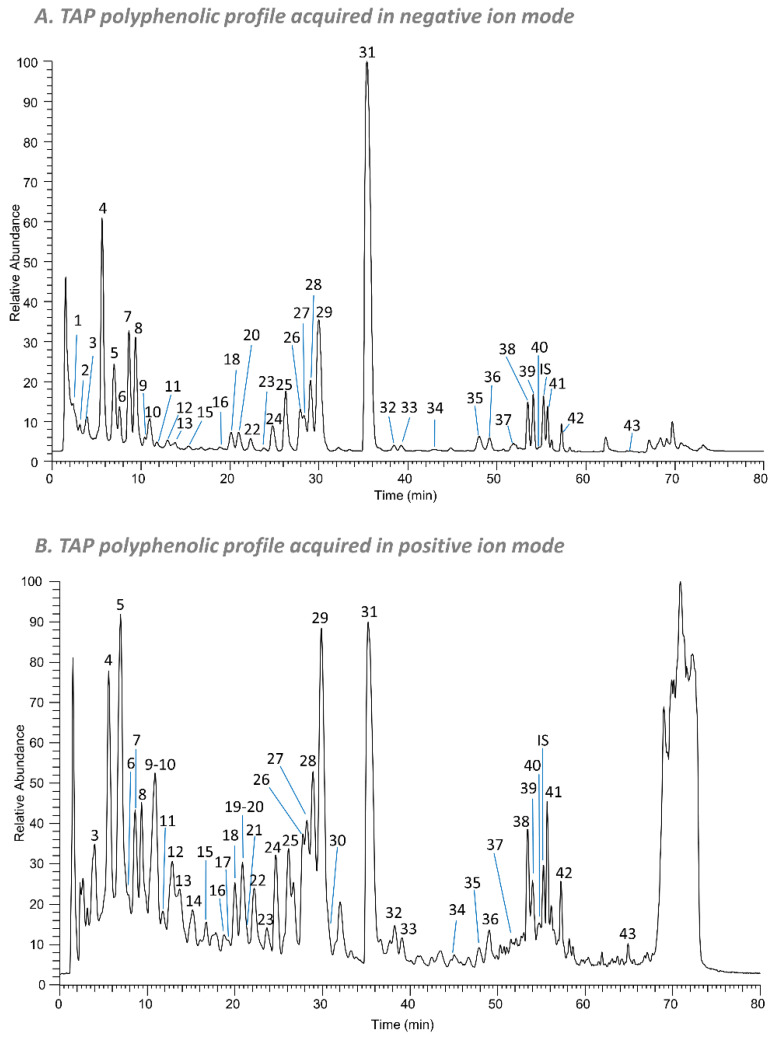
Total ion currents (TICs) of PAT fraction acquired in (**A**) negative ion mode and (**B**) positive ion mode. Identified peaks are numbered progressively, according to the elution order, and their identity or putative identity is shown in Table S1 of the Supplementary Materials with the experimental mass, MS/MS fragments and identification method. IS: internal standard.

The qualitative profile of polyphenol components of TAP was firstly evaluated by a targeted and untargeted metabolomic approach by HPLC-HRMS in negative and positive ion mode. Figure 2 shows the chromatograms of the extract as total ion current (TIC) recorded in negative (A) and positive (B) ion mode where the identified peaks are numbered progressively (co-eluting peaks share the same number), according to the elution order. The peak of the internal standard (Trolox) is indicated by “IS”. A total of 68 compounds were identified, 68 of which are polyphenols, 52 by the targeted approach and 16 by the untargeted approach.

The 52 compounds identified or putatively identified with the targeted analysis are listed in Table S1 of the Supplementary Materials; for each identified compound, the relative peak number (Figure 2), the retention time (RT), the experimental mass (as [M−H]^−^, [M+H]^+^, [M+2H]^2+^ or [M+Na]^+^), the mass accuracy (Δppm), MS/MS fragments and identification method are reported. Thirty-nine compounds were detected in both polarities, seven in negative ion mode and six in positive ion mode. Among the 52 components, 20 are phenolic and organic acids, 11 are flavanols, 10 are flavonols, 5 are flavanones, 4 are dihydrochalchones, 1 is a flavone (luteolin) and 1 is a triterpenoid (euscaphic acid). Thirteen compounds were confirmed by means of in-house available pure standards: three phenolic acids, protocatechuic acid, caffeic acid and *p*-coumaric acid; two flavanols, catechin and epicatechin; two flavanones, naringenin glucoside and naringin; four flavonols, quercetin-3-O-galactoside, quercetin-3-O-rhamnoside, quercetin and kaempferol; two dihydrochalcones, phlorizin and phloretin; one flavone, luteolin. For the compounds for which the standard was not available (39 molecules), a putative identification was carried out by matching the accurate mass, the isotopic pattern and the fragmentation pattern with data reported in the literature. Among phenolic acids, the glucoside forms of protocatechuic, ferulic, caffeic and coumaric acids, and also quinic acid esters of caffeic and coumaric acids were identified. Flavanols are represented in the monomeric forms (catechin and epicatechin) up to the nonamer oligomer, detected as [M+2H]^2+^. Naringenin and eriodictyol derivatives (and aglycones) were the flavanones identified in the extract. The untargeted analysis allowed the putative identification of 16 compounds (Table S2 of Supplementary Materials): nine flavonols, three phenolic acids, one flavanone, one dihydrochalchone and two lipids. As an example, 3-(benzoyloxy)-2-hydroxypropyl glucopyranosiduronic acid (compound **11**) was tentatively annotated, firstly by calculating the possible molecular formula with the QualBrowser tool of Xcalibur (mass tolerance of 5 ppm and using C, H, O, N, S as possible atoms). The resulting formulae were searched in databases and in the literature and we found a match with 3-(benzoyloxy)-2-hydroxypropyl glucopyranosiduronic acid (C_16_H_20_O_10_, 0.037 ppm, in negative ion mode) [52]. The MS/MS spectrum was also compared: the main fragment was at *m/z* 249 in negative ion mode deriving from the loss of benzoic acid (−122 Da). Glycosides of quercetin, methoxyquercetin (patuletin, detected with the untargeted approach) and kaempferol are the most representative flavonols identified, but they were also present as aglycones, and a coumaroylglucoside derivative of quercetin was annotated with the untargeted approach. Methoxyquercetin (patuletin, compound **36**) was putatively identified through HMDB database: the molecular formula C_16_H_12_O_8_ (0.107 ppm in negative ion mode; 1.862 ppm in positive ion mode) and MS/MS fragments at *m/z* 285, 209 and 181 (in negative ion mode) matched with those found in the database, and in addition, we detected an additional fragment at *m/z* 316, corresponding to the loss of a methyl group (−15 Da). We detected also five patuletin derivatives: three hexoside isomers (compounds **20**, **22** and **23**) at 20.8, 22.5 and 23.8 min, tentatively annotated with PubChem; one patuletin pentoside derivative (compound **27**), tentatively identified through the matching of the molecular formula (C_21_H_20_O_12_, −0.179 ppm and −1.591 ppm in negative and positive ion mode, respectively); and characteristic fragments of patuletin aglycone at *m/z* 331 and 316 due to a neutral loss of −132 Da, corresponding to a pentoside moiety and a subsequent loss of a methyl moiety. Similarly, patuletin rhamnoside (compound **29**) was annotated through the molecular formula (C_22_H_22_O_12_, 0.161 ppm and −2.066 ppm in negative and positive ion mode, respectively) and the MS/MS fragments (at *m/z* 331 and 316) deriving from the neutral loss of a rhamnoside moiety (−146 Da) and a subsequent loss of a methyl (−15 Da). Patuletin is a methoxy derivative of quercetin which, among the bioactivities reported in the literature, was found effective in the reduction of serum TNF-α in a rodent model of adjuvant-induced arthritis [53]. CFM-ID online software gave for compound **38** eriodictyol 7-(6-trans-*p*-coumaroylglucoside) as the best match, using the compound identification tool which compares the molecular ion (mass tolerance of 5 ppm) and the MS/MS fragments to data present in online databases. Coumaroyl glucosides seems to show a higher antioxidant effect with respect to the relative glucoside derivative as reported by Li X. et al. [54]: in TAP extract, we found eriodictyol 7-(6-trans-*p*-coumaroylglucoside) and quercetin 3-(3-*p*-coumaroylglucoside) (putatively identified with HMDB). The phloretin derivatives typically identified in apples were also found, with a new putative annotation (through HMDB) of hydroxyphloretin glucoside (peak **25**) detected with the untargeted approach. The untargeted approach also revealed two lipids (peaks **41** and **42**), annotated as (10E,15Z)-9,12,13-trihydroxy-10,15-octadecadienoic acid and trihydroxy-octadecenoic acid.

### 3.2. Quantitative Analyses (Total Polyphenols and HPLC)

The total polyphenol content was determined both by spectrophotometry and HPLC analysis as reported in the Section 2. Overall, the results derived by these two methods are superimposable, being 24.14 ± 1.58 and 27.97 ± 0.68 mg/100 mg, as determined by the Folin–Ciocalteu colorimetric test and HPLC analysis, respectively.

By considering the values reported by Sun et al., who found that the total extractable phenolic content from fresh apples is between 110 and 357 mg/100 g [55], we can say that the preparation of the TAP extract resulted in a significant increase in the recovery yield of the polyphenolic fraction.

### 3.3. Direct Radical Scavenging Activity (DPPH)

The direct radical scavenging activity was evaluated by the DPPH assay and the results are reported in Table 1 and expressed as IC_50_. The lower the value obtained, the higher the direct radical scavenging activity. The TAP extract was found to exert a significant radical scavenging activity, which was found to be significantly higher than the reference compounds when expressed on the basis of the polyphenol content as determined by HPLC.

### 3.4. NRF2 Activation and Anti-Inflammatory Activity

**Figure 3:**
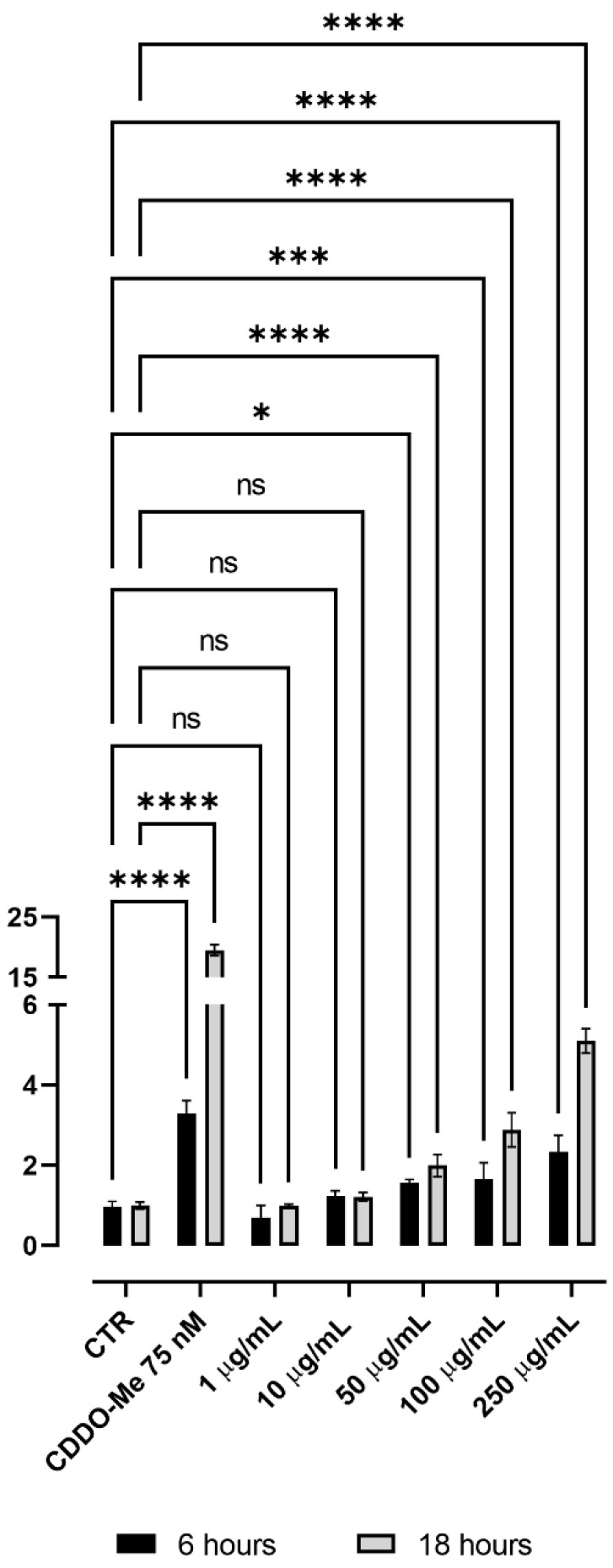
Dose-dependent effect of TAP on NRF2 nuclear translocation. NRF2 activation was tested using NRF2/ARE Responsive Luciferase Reporter HEK293 stable cell line incubated for 6 and 18 h with TAP in a concentration range between 1 and 250 μg/mL. CDDO-Me 75 nM was used as a positive control. Statistical analysis was calculated by two-way ANOVA with Dunnett’s multiple comparison test, with individual variances computed for each comparison (* *p* < 0.05, *** *p* < 0.001, **** *p* < 0.0001, ns: not significant).

We first tested the effect of TAP on cell viability using the MTT assay up to a concentration of 250 μg/mL. Cell viability was found to be higher than 95% at all the tested doses (data not shown). Figure 3 reports the dose-dependent effect of TAP on NRF2 activation after 6 and 18 h of incubation in a concentration range between 1 and 250 μg/mL. After 6 h, the effect started to be significant at a concentration of 50 μg/mL and induced a 2.3-fold increase at a 250 μg/mL concentration. The fold increase was higher after an incubation time of 18 h to reach more than a 5-fold increase at the highest tested dose.

**Figure 4:**
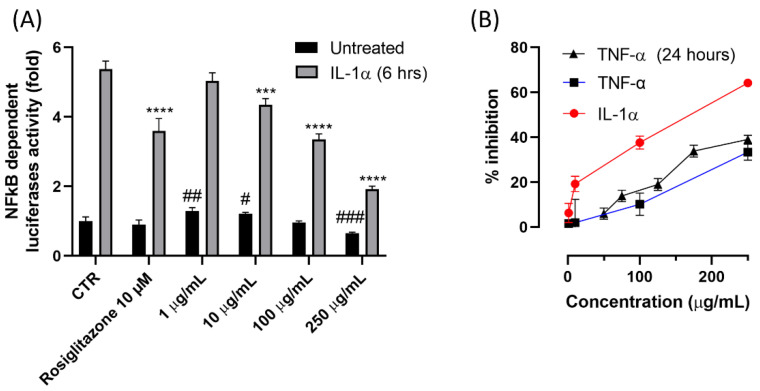
Dose-dependent anti-inflammatory activity of TAP. Activity was tested in R3/1 with the luciferase gene reported for NF-κB nuclear translocation. (**A**) The NF-κB-dependent luciferase activity in cells incubated in the absence (black columns) and presence (gray columns) of IL-1α and treated with TAP in a 1–250 μg/mL concentration range. Rosiglitazone 10 μM was used as a positive control. (**B**) The dose-dependent effect of TAP on the NF-κB-dependent luciferase activity of cells challenged for 6 h with IL1-α, and for 6 and 24 h with TNF-α, (*** *p* < 0.001, **** *p* < 0.0001—# *p* < 0.05, ## *p* < 0.005, ### *p* < 0.001).

The anti-inflammatory activity of TAP extract was then tested in the same concentration range (1–250 μg/mL) using R3/1 NF-κB cells and two different inflammatory inducers: TNF-α and IL-1α. The results are summarized in Figure 4. (A) shows the NF-κB-dependent luciferase activity in cells incubated in the absence (black columns) and presence (gray columns) of IL-1α and treated with TAP. Results are reported as luciferase fold increase with respect to untreated cells. IL-1α induced more than a 5-fold increase in NF-κB-induced luciferase, which was dose-dependently reduced by TAP incubation. Luciferase activity as not affected by TAP in the absence of IL-1α. TAP was then tested using TNF-α as a stimulus (Figure 4B), and in this condition, it was found to dose-dependently reduce the NF-κB-dependent luciferase activity after both 6 and 24 h of incubation. The TAP anti-inflammatory activity was greater when IL-1α was used as an inflammatory stimulus with respect to TNF-α.

### 3.5. Quantitative Proteomic Studies

The effect of TAP on the proteome of control cells and cells stimulated with TNF-α was then studied by using two different quantitative proteomic approaches, SILAC and label-free, and the following four different experimental groups: control cells, cells stimulated with TNF-α, and cells incubated in the absence and presence of TAP.

#### 3.5.1. SILAC Proteomic Studies

**Figure 5:**
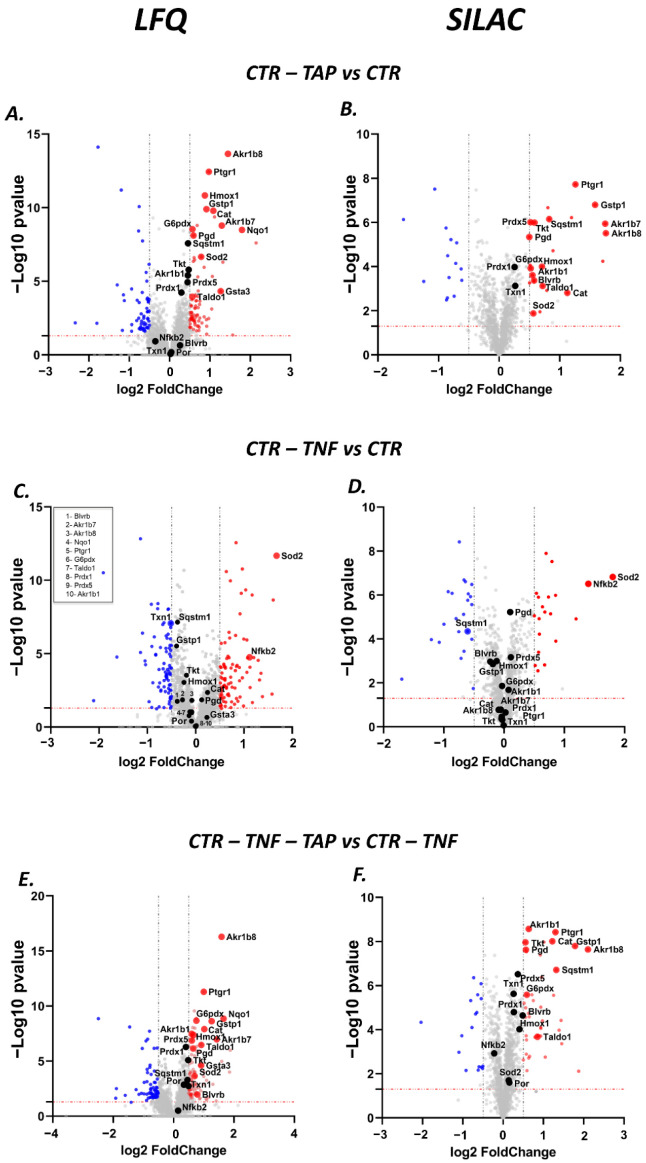
Volcano plot of the LFQ and SILAC analyses. The two columns represent the LFQ (left) or SILAC (right) analyses. In the first row, the two volcano plots (**A**,**B**) illustrate the analyses performed to understand the impact of TAP extract on the cell proteome. On the second row, (**C**,**D**) show the volcano plot for the comparative condition achieved by treating cells with TNFα. Lastly, (**E**,**F**) are the volcano plots showing the differential proteome expression for the inflamed cells treated with TAP extract. In each volcano plot, the most representative proteins identified in the various analyses are labeled in red if up-regulated (*p* value < 0.05, log2 *Fold-change* > 0.5), in blue if down-regulated (*p* value < 0.05, log2 *Fold-change* < −0.5) and in black when they do not fall into the two aforementioned conditions. A more comprehensive analysis of the log2 *Fold-change* values is available in Table 2.

Figure 5 shows the volcano plots obtained from the three comparative analyses: (i) cells incubated in the absence (CTR) and presence of TAP (CTR-TAP), (ii) cells incubated in the absence (CTR) and presence of TNF-α (CTR-TNF); (iii) cells stimulated with TNF-α and incubated in the absence (CTR-TNF) and presence of TAP (CTR-TNF-TAP). The analyses were conducted through two orthogonal proteomic approaches, SILAC and the label-free method.

**Figure 6:**
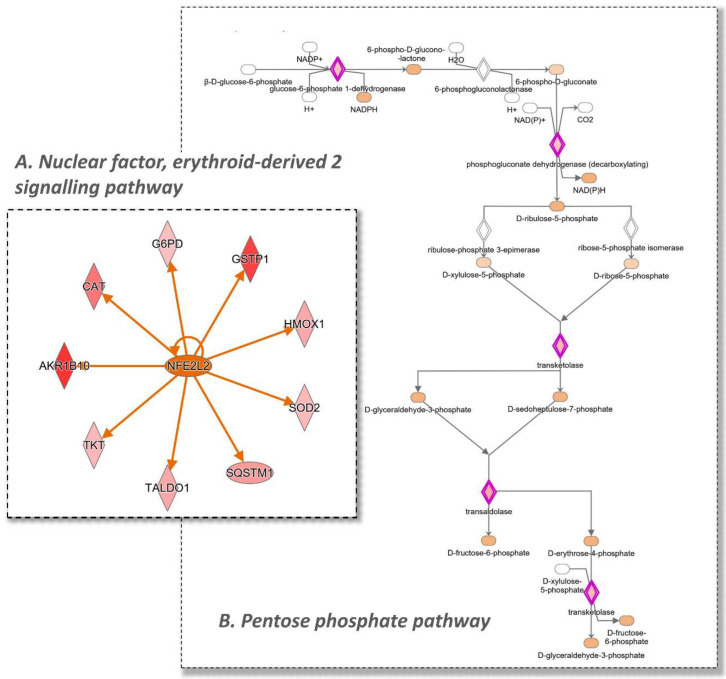
IPA analysis of the effect of the extract on the cell proteome. (**A**) illustrates that the differentially regulated proteins in the analysis are consistent with an NRF2 activation. (**B**) illustrates the Pentose Phosphate Pathway; in red are the proteins that were found to be up-regulated by TAP, resulting in a increment in the NADPH pool in the cell. Color legend: red represents the increased genes, green the decreased (not present in the figure). The intensity of the color is related to the intensity of up- or down-regulation. The orange line leads to activation and a blue line (not present in the figure) leads to inactivation. The yellow line indicates that findings that are not consistent with the proteomics results obtained.

The first experimental comparison analysis permits the evaluation of the general effect of TAP on the cell proteome in homeostatic conditions. From the SILAC experiment, 24 and 14 proteins were found to be up- and down-regulated, respectively, while 1235 were found to be unchanged. Ingenuity pathway analysis identified two main up-regulated pathways: the nuclear factor, erythroid-derived 2 signaling pathway with a z-Score of +2.9 (Figure 6A), and the pentose phosphate pathway with a z-Score of +2.0 (Figure 6B).

In particular, the up-regulated enzymes involved in the first pathway include HMOX1, CAT, GSTP1, AKR(1–7), NQO1 and SQSTM1, while for the second one, TKT, PGD, G6PDX and TALDO1 are identified. In addition to these proteins, PTGR1 and BLVRB were also found to be up-regulated, as reported in the volcano plots (Figure 5). PTGR1 is an NADPH-dependent oxidoreductase which is found to be induced by NRF2 activators [56] and directly regulated by NRF2 [57]. BLVRB is found to be an NRF2 target gene and together with HMOX is critical in the heme metabolism [58] (Figure 11A).

The volcano plot relative to the second experimental comparison analysis (CTR-TNF vs. CTR, Figure 5C,D) mainly identifies two overexpressed proteins involved in inflammation, NF-ΚB2 and SOD2. As expected, the ingenuity pathway analysis identifies, with a z-Score of 1.8, the TNF-α as up-stream regulator (data not shown).

The volcano plot displaying the third comparison analysis (CTR-TNF-TAP vs. CTR-TNF, Figure 5E,F) reports that TAP treatment down-regulates the two proteins over-expressed in the inflammatory conditions (NF-ΚB2 and SOD2), while the two main pathways up-regulated by the TPA treatment were confirmed to be the NRF2 and the pentose phosphate pathway.

**Figure 7:**
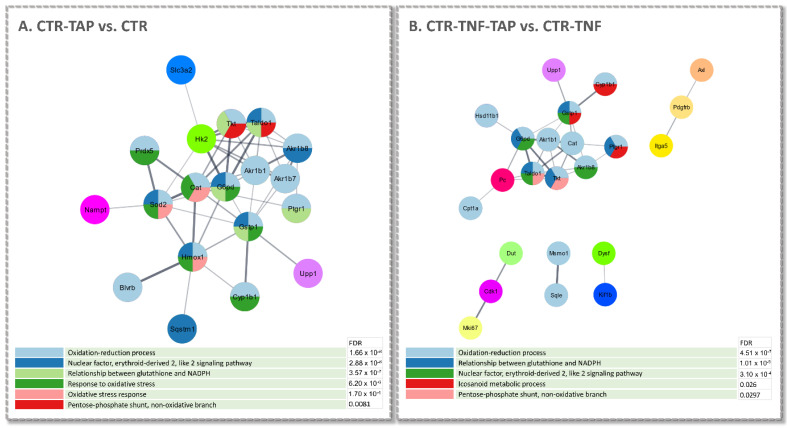
Cytoscape and STRING analysis of the up-regulated gene products determined by SILAC. The panels illustrate the protein–protein interaction networks obtained by STRING in the CTR-TAP vs. CTR (**A**) and CTR-TNF-TAP vs. CTR (**B**) conditions. The color/s of each node (gene product) reflect/s the functional enrichment analysis performed; the color code describing the enriched biological processes with the corresponding FDR value is shown below each network. In both analyses, the singlets were left out.

Finally, protein–protein interaction network analysis using the Cytoscape interface in STRING confirmed the modulation of a protein set involved in the biological processes/pathways already highlighted in IPA, as a result of TAP treatment (Figure 7A,B). Assigned GO annotations lead firstly to confirmation of the activation of the NRF2 and pentose phosphate pathways and the involvement of several oxidoreductive enzymes as well as of proteins involved in the metabolism of glutathione (GSH). It should be taken into account that STRING–Cytoscape network analysis does not distinguish between up- and down-regulated proteins but only considers significant expression modulation.

#### 3.5.2. Label-Free Quantitative Proteomic Studies

**Figure 8:**
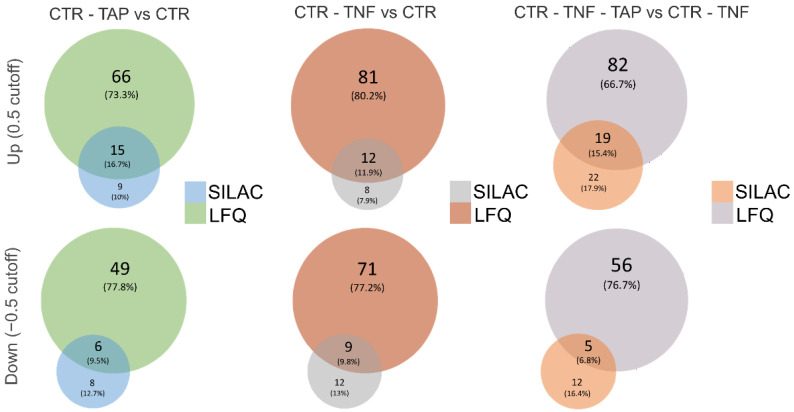
Venn diagrams showing technical differences in terms of the number of significantly (log ratio cutoff ± 0.5) up- (first line diagrams) and down-regulated (second line diagrams) proteins identified by the two approaches in the three comparison analyses (CTR-TAP vs. CTR; CTR-TNF vs. CTR; CTR-TNF-TAP vs. CTR-TNF). Additionally, the percentage distribution of identifications in the two approaches and the percentage of common assignments are shown for each diagram.

The label-free quantitative proteomic study, through the use of a Fusion MS analyzer, identified a larger number of proteins with respect to those identified by the SILAC approach, which was based on an LTQ Orbitrap system. In particular, LFQ identified 2340 more proteins than SILAC in the CTR-TNF vs. CTR mixture, 2412 more in the CTR-TAP vs. CTR mixture and 2259 more in the CTR-TNF-TAP vs. CTR-TNF mixture. As shown by the volcano plots relative to the three experimental conditions’ comparison, depicted in Figure 5 (lower panels), besides a larger number of identified proteins, the LFQ approach also permitted the identification of more up- and down-regulated proteins in all three conditions. Details about the comparison of the number of up- and down-regulated proteins identified by the two approaches in the three experimental groups are shown by Venn diagrams in Figure 8.

**Figure 9:**
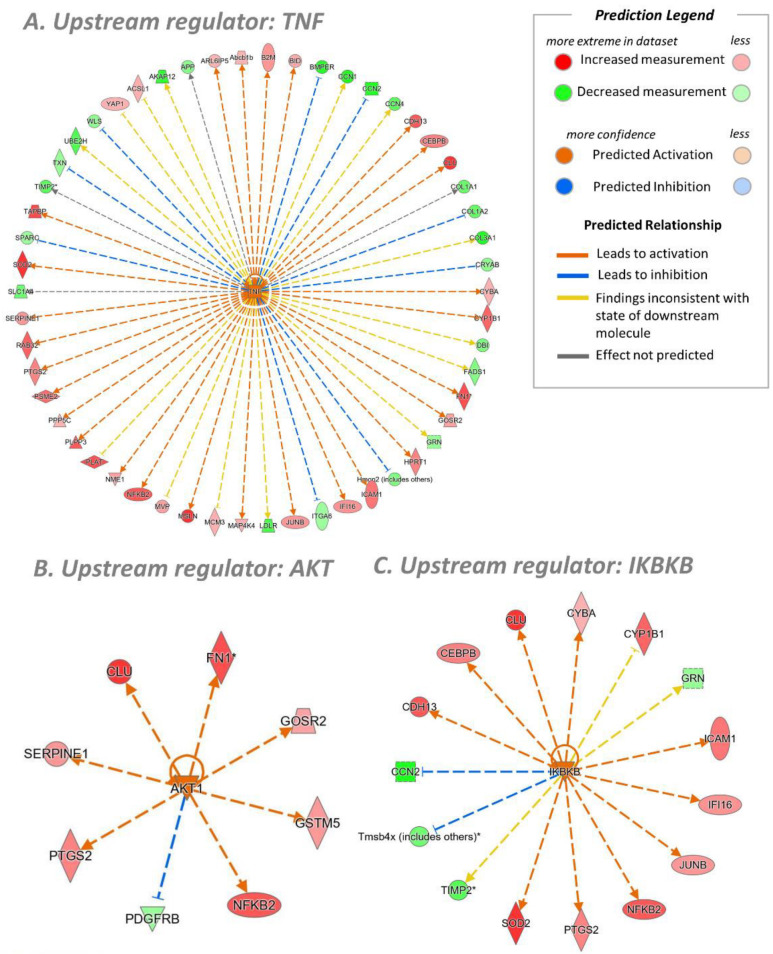
Upstream regulators determined in the CTR-TNF vs. CTR condition by IPA analysis (LFQ). (**A**) The wheel of proteins involved in the TNFα stimuli. (**B**,**C**) The wheels of proteins associated with AKT and IKBKB as upstream regulators. Above is the prediction legend (IPA) essential for understanding networks. * multiple isoforms.

Pathway analysis (IPA) of the LFQ results, besides confirming the results achieved by the SILAC approach, identified further regulated pathways. As shown in Figure 9, a better description of the NF-κB activation was observed because two upstream regulators were found to be compatible with the over-expressed proteins, AKT (protein kinase B, Figure 9B) and IKBKB (inhibitor of nuclear factor kappa-B kinase subunit beta, Figure 9C). AKT regulates the transcriptional activity of NF-κB by inducing phosphorylation and the subsequent degradation of the inhibitor of κB (IκB). IKBKB phosphorylates the inhibitor in the inhibitor/NF-κB complex, causing dissociation of the inhibitor and activation of NF-κB. As a result, the number of proteins identified in the TNF-a pathway significantly increases. Besides AKT and IKBKB, another obvious upstream regulator is reported in Figure 9A, the TNF pathway, which simply confirms the effectiveness of the method used to induce inflammation.

Regarding the CTR-TAP vs. CTR comparison, the activation of the NRF2 pathway by TAP, as already evidenced by the SILAC approach, was confirmed and the number of proteins involved in the signaling was greatly extended. Furthermore, TAP treatment was associated with a reduction in ferroptosis, apoptosis and oxidative stress (data not shown).

**Figure 10:**
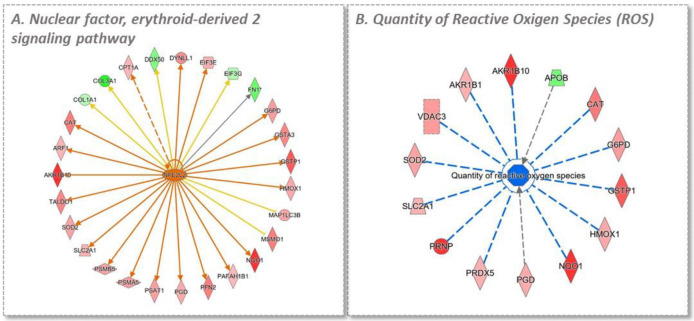
IPA analysis in the CTR-TNF-TAP vs. CTR-TNF condition. (**A**) illustrates the different regulated proteins present in the analysis which correlate with NRF2 activation. (**B**) illustrates the effect that the differentially regulated proteins have on the quantity of reactive oxygen species, confirming a trend of reduction in oxidative stress. The prediction legend (IPA) is reported in Figure 9.

Regarding the third group, CTR-TNF-TAP vs. CTR-TNF, the LFQ approach permitted a better description of the upstream regulators relative to a reduction in oxidative stress and NRF2 pathway due to the increased number of the identified proteins involved in the pathways (Figure 10).

## 4. Discussion

Epidemiological and intervention studies indicate that consumption of apples and derivatives, such as apple juice, exert beneficial effects relating to some human diseases including CVD and related events, e.g., cancer and diabetes. Some of the health effects, and in particular, the antioxidant and anti-inflammatory activities, have been attributed to the polyphenol fraction, as also confirmed by pre-clinical studies. Hence, there is a growing interest in the use of apple polyphenols as health care products, as well as on their production with green methods. Apple polyphenols are contained in large quantities in thinned apples which represent a large waste material of the apple chain. Based on these premises, the aim of the present study was to evaluate thinned apples as a source for the isolation of bioactive apple polyphenols, effective as anti-inflammatory and antioxidant compounds to be used in health care products. To reach this goal, we first set up a scalable procedure for polyphenol isolation from thinned apple. The polyphenol content of the purified fraction obtained by absorption resin accounted for 24%, as determined by colorimetric analysis, and almost 28% by HPLC, indicating the suitability of the isolation process. The isolated polyphenols were then fully identified by a targeted and untargeted LC-HRMS approach which is, to our knowledge, the first qualitative profile of polyphenols from thinned apples.

The targeted and untargeted metabolomics approaches allowed us to identify a total of 68 polyphenols belonging to the six polyphenolic classes. Moreover, 16 compounds were found in the TAP fraction, though not yet reported in apple or derivatives. All the characteristic polyphenols identified in harvested apples as reported in the most recent literature were identified.

The biological evaluation of the extract followed the qualitative characterization; TAP was first evaluated in two cell models in order to evaluate the antioxidant and anti-inflammatory properties of the extract, the two main activities ascribed to apple polyphenols as reported by the most recent literature. The antioxidant activity was firstly studied by the DPPH assay, which demonstrated a greater radical scavenging activity of the polyphenol fraction with respect to reference compounds, i.e., Trolox and ascorbic acid. For several years, the in vivo antioxidant activity of plant polyphenols has been explained by considering their direct radical scavenging activity towards ROS and carbon centered radicals. However, as pointed out by Forman and Ursini [59], kinetic constraints indicate that in vivo scavenging of radicals is ineffective in antioxidant defense (except for vitamin E towards peroxyl radicals). Instead, enzymatic removal of non-radical electrophiles, such as hydroperoxides, in two-electron redox reactions, is the major antioxidant mechanism. Based on this overview, the major mechanism of action for nutritional antioxidants, and in particular, for plant polyphenols such as those contained in TAP, is the paradoxical oxidative activation of the NRF2 (NF-E2-related factor 2) signaling pathway, which maintains protective oxidoreductases and their nucleophilic substrates. As stated by Ursini, the maintenance of the “Nucleophilic Tone” by a mechanism called “Para-Hormesis” provides a means for regulating physiological non-toxic concentrations of the non-radical oxidant electrophiles that boost antioxidant enzymes, damage removal and repair systems (for proteins, lipids and DNA) at the optimal levels consistent with good health [59].

Hence, based on this new vision of the antioxidant activity of natural antioxidants, as a next step, we tested the ability of TAP to act as an indirect antioxidant by activating the NRF2 pathway.

Results well indicate that TAP dose-dependently activates the NRF2 pathway, as determined by measuring the activity of luciferase, which is the product of the gene reporter activated by the nuclear binding of NRF2. Activation of NRF2 by TAP can be firstly ascribed to the polyphenol compounds bearing an ortho-diphenol moiety which is oxidized to the corresponding quinone, which, being an electrophilic compound, reacts with the thiols of KEAP1, thus releasing NRF2, which then translocates into the nucleus. TAP contains a set of polyphenols containing the catechol moiety, including derivatives of caffeic acid, and quercetin. TAP also contains phenols or methoxy derivatives, such as derivatives of coumaric acid, phloretin and naringenin, which can also be NRF2 activators, but in this case, metabolic activation is required to form a di-phenol moiety. In particular, the metabolic activation of phenols requires the insertion of a hydroxyl group in ortho or para position mediated by the cytochrome enzymes, as found for resveratrol [60], while a CYP mediated O-demethylation activates methoxy derivatives, as reported for silybin [61]. Such metabolic reactions usually occur in the liver tissue and hence are unlikely to occur in the cell model used in the in vitro assay. Hence, we presume that the potency of NRF2 activation is underestimated and that presumably it is potentiated in vivo by metabolic activation occurring in the organism, as well as by the microbiota in the gastrointestinal tract.

NRF2 activation paralleled the dose-dependent anti-inflammatory activity, as demonstrated in the tested cell model (luciferase gene reporter for NF-κB nuclear translocation), and using two different pro-inflammatory agents, IL1-α and TNF-α. A strict interdependence between oxidative stress and inflammation and between the two transcriptional factors involved, NF-κB and NRF2, is well established [62,63]. When oxidative stress appears as a primary disorder, inflammation develops as a secondary disorder and further enhances oxidative stress. On the other hand, inflammation as a primary disorder can induce oxidative stress as a secondary disorder, which can further enhance inflammation [64]. Based on this evidence, a well-established strategy to block the inflammatory chronic condition consists of inhibiting oxidative stress, and in turn, the best approach to inhibit oxidative stress is to activate the nucleophilic tone by inducing NRF2 nuclear translocation.

Hence, the anti-inflammatory activity of TAP can be first of all ascribed to the NRF2-2-dependent antioxidant effect. To gain a deeper insight into this mechanism, and in particular, to confirm the activation of antioxidant enzymes and to search for other enzymes involved in the anti-inflammatory activity, a proteomic investigation was then carried out.

Quantitative proteomic studies were carried out using the cell line with the gene reporter for NF-κB, used for the anti-inflammatory activity evaluation, and TNF-α as an inflammatory inducer. Two orthogonal proteomic approaches were applied, namely, a label-free method, using a Fusion MS analyzer, and a stable isotope in vivo labeling method, i.e., SILAC, coupled to a LTQ Orbitrap system. In general, the label-free approach identified many more proteins with respect to SILAC and was revealed to be more efficient in the identification of up- and down-regulated pathways. These differences could be easily explained by considering the intrinsic potential in terms of sensitivity and resolution characteristics of the mass analyzers used for each of the experiments. Besides the different performances, the two approaches identified the same modulated pathways, although with a different number of proteins, making the results more robust, as confirmed by two orthogonal approaches. This very interesting technical aspect is particularly evident in the following table (Table 2), which shows the experimentally calculated fold-change values for each key protein identified, whose expression is significantly modulated in the comparative analysis between the tested conditions.

The first relevant observation emerging from the comparison of the calculated fold-change values in the different conditions tested is the apparent shift in the expression of some of the proteins involved in the NRF2 and pentose phosphate pathways. A fitting example could be the HMOX1 expression modulation, having a negative fold-change (LFQ: −0.2424, SILAC: −0.1233) in the first comparison, meaning that inflammation causes its down-regulation, and showing a significant up-regulation when the cells are treated with TAP extract, in both conditions.

Targeting a more comprehensive biological significance, rather than investigating individual key proteins, the global effect of TAP treatment in physiological conditions (CTR), to identify the set of genes whose expression is variably modulated by treatment, was evaluated. As expected, many up-regulated genes involved in the NRF2 pathway activation were detected, particularly antioxidant enzymes, or those directly involved in the detoxification of peroxides and in two-electron redox reactions, e.g., SOD, CAT, GXP and redox proteins such as thioredoxins. Furthermore, a set of enzymes required for the synthesis of GSH and NADPH, co-factors for the enzymes involved in the antioxidant and redox regulation, were also identified. GCLC, GSTA3 and GSTP were the identified up-regulated enzymes involved in the GSH synthesis and metabolism while the enzymes belonging to the oxidative pentose-phosphate pathway are those up-regulated for NADPH synthesis, such as malic enzyme-1 (ME-1), isocitrate dehydrogenase-1 (IDH1), glucose-6-phosphate dehydrogenase (G6PDX) and 6-phosphogluoconate dehydrogenase (PGD).

The crucial finding is that TAP treatment over-expresses the inducible isoform of heme oxygenase (HMOX1), a well-established immunomodulator [65,66,67]; it has been proven that the induction of HMOX1 protects against the cytotoxicity caused by oxidative stress and apoptotic cell death, making HMOX1 an appealing target for the treatment of several chronic inflammatory diseases, including osteoporosis [68], cancer [69], acute kidney injury [70], retinal pigment epithelium degeneration [71] and Parkinson’s disease [56,67].

**Figure 11:**
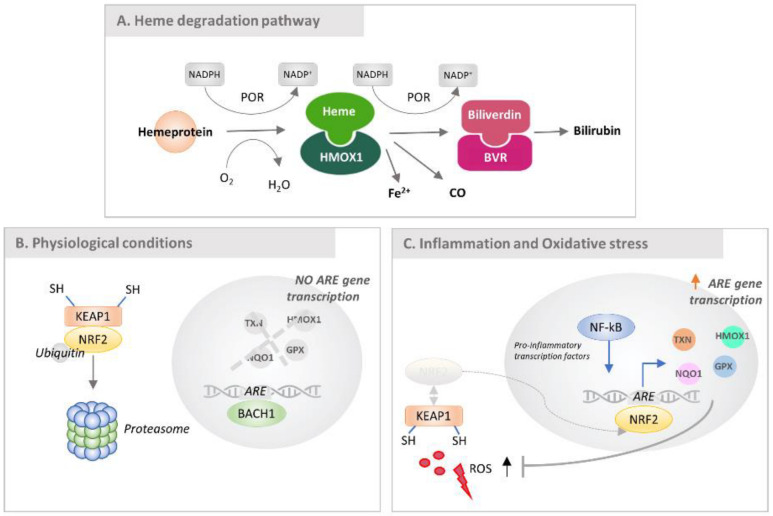
Signaling pathways in which HMOX1 is involved. (**A**) Heme degradation pathway promoted by HMOX1 occurring in the cytosol of the cell. Heme degradation occurs in the cytosol by the enzyme HMOX1; the reaction involves cytochrome P450 reductase, NADPH as cofactor and O_2_, leading to the production of NADP^+^, and an equimolar amount of biliverdin, carbon monoxide and iron (Fe^2+^). Specifically, biliverdin can be further converted to bilirubin by the enzyme biliverdin reductase (BVR). (**B**) Regulation of HMOX1 expression under homeostatic conditions. NRF2 is bound to KEAP1, ubiquitinated and targeted for proteasomal degradation. BACH1 binds the antioxidant response element (ARE) at the promoter of HMOX1, inhibiting transcription of the gene. (**C**) Regulation of HMOX1 expression during inflammation and oxidative stress. Increased intracellular heme levels lead BACH1 to dissociate from ARE, and reactive oxygen species (ROS), interacting with cysteine residues on KEAP1, cause dissociation from NRF2, which in turn migrates into the nucleus, binds ARE and promotes transcription of HMOX1 (adapted from [67]).

In physiological conditions, HMOX1 is involved in rate-limiting heme degradation using NADPH–cytochrome P450 reductase (POR) and oxygen to generate linear tetrapyrrole biliverdin (further converted into linear tetrapyrrole bilirubin by the enzyme biliverdin reductase, BLVRB), ferrous iron (Fe^2+^) and carbon monoxide (CO) (Figure 11A). The enzymatic heme-degradation by-products are known to be the main cause of the beneficial protective effects promoted by HMOX1; bilirubin, in particular, is one of the most potent antioxidant and is particularly effective in protecting against lipid peroxidation.

HMOX1 can be up-regulated in response to numerous different stress stimuli, including various pro-oxidant and pro-inflammatory mediators; most of them do not interact directly with the transcription factors, but activate them via intermediate signaling pathways. HMOX1 expression is largely under the control of NRF2, a well-known binder of the ARE (antioxidant response element), aimed at promoting many antioxidant genes, including HMOX1. Under homeostatic conditions, NRF2 is bound to Kelch-like ECH-associated protein 1 (KEAP1), ubiquitylated and targeted by the proteosome for degradation (Figure 11B); meanwhile, BACH1 binds to ARE and inhibits gene transcription. An increased level of reactive oxygen species (ROS) during cell stress implies a concomitant KEAP1-NRF2 dissociation due to their reactivity against thiols in KEAP1; in turn, NRF2 translocates to the nucleus and binds the ARE to promote transcription of HMOX1 (Figure 11C). Basically, NRF2, KEAP1 and BACH1 constitute an intricate feedback system enabling cells to respond to oxidative stress ending at the up-regulation of HMOX1.

The pathway activation in this study is supported not only by the significant modulation of HMOX1 but also by the up-regulation of BLVRB and POR (NAPH-Cytochrome P450 reductase), which, although not significantly up-regulated (fold-change: 0.340528912, *p*-value n.s.), exactly matches the trend of the other associated gene products. In addition, activation of the pentose phosphate pathway leads to a plausible increase in circulating NADPH, a cofactor of both POR and BLVRB (Table 2).

The phenotypic effect described here, in terms of protein expression induced by TAP treatment, is completely concordant when comparing CTR-TNF-TAP to CTR-TNF conditions. The protective effect manifested, such as the triggering of the two pathways of interest, is perhaps even more evident looking at the volcano plots shown in Figure 5A,B: AKR1B1, AKR1B8, BLVRB, CAT, G6PDX, GSTP1, HMOX1, PGD, PTGR1, SQSTM1, TALDO1 and TKT (listed alphabetically) switch to a fold-change value indicating significant up-regulation, explaining the anti-inflammatory activity overall.

Besides the up-regulated set of NRF2-dependent pathways, another key point is the regulation of TAP towards the increased content of NF-κB in cells exposed to TNF-α with respect to control (Figure 5C,D), and the overexpression of a set of proteins which are known to be overexpressed by the TNF-signaling. Hence, NF-κB, in the cell model used, besides being activated and translocated at the nuclear level, as evidenced by the gene reporter, is also overexpressed, thus sustaining the inflammatory process. As stated above, TAP treatment of the inflamed cells not only inhibits NF-κB activation but also its overexpression: NF-κB2, together with SOD2, returns to homeostatic conditions, even if the fold-change was not significantly different from zero (Figure 5E,F). An interesting aspect to stress is that NF-κB, together with AP-1 (Figure 11C), can also bind the HMOX1 promoter to enhance its expression; indeed, some instances of HMOX1 up-regulation in vitro have been shown to be dependent on or correlated with NF-κB or AP-1 expression [72].

## 5. Conclusions

In conclusion, thinned apples represent a valuable source of apple polyphenols whose qualitative profile was fully elucidated by targeted and untargeted metabolomic approaches. The anti-inflammatory and antioxidant activities of TPA were first evaluated by cell-based assays using engineered cells with gene reporters for NRF2 and NF-κB nuclear translocation. Quantitative proteomic studies were then used to deeply investigate the molecular mechanisms involved in the proven biological activity. Two different proteomic approaches, i.e., label-free and SILAC, both confirmed TAP ability in improving the antioxidant cellular defense, by activating the NRF2 signaling pathway which up-regulates protective oxidoreductases and their nucleophilic substrates. A clear increase in enzymatic antioxidants together with the up-regulation of the heme-oxygenase explain the anti-inflammatory effect of TAP. Taken together, these results suggest that thinned apples can be effectively considered a valuable source of apple polyphenols to be used in health care products to prevent/treat oxidative and inflammatory chronic conditions.

## Figures and Tables

**Table 1:** Direct radical scavenging activity of TAP extract, TAP expressed on the basis of polyphenol content, Trolox, and ascorbic acid. Results are reported as mean ± standard deviation.

Sample	Radical Scavenging Activity IC_50_ µg/mL (Mean ± SD)
TAP TAP (expressed on polyphenol content)	11.4 ± 1.1 3.2 ± 0.3
Trolox	5.0 ± 0.3
Ascorbic acid	3.9 ± 0.05

**Table 2:** Key proteins identified by quantitative proteomics analysis; fold-change values calculated by each of the experimental approaches used are reported by gene product. The fold-change values are classified according to the comparative analysis of the conditions tested. ND indicates a not-detected protein identification with the corresponding proteomic approach. The value 0 indicates a fold-change not significantly different from zero. Each fold-change set, reported per gene product and per proteomic approach, is colored on a green (minimum value) to light orange (maximum value) color scale according to the conditional formatting function of excel. The gene products involved in the nuclear factor erythroid-derived 2 signaling pathway are highlighted in light violet and those involved in the pentose phosphate pathway in light blue.

	*CTR-TNF* vs. *CTR*		*CTR-TAP* vs. *CTR*		*CTR-TNF-TAP* vs. *CTR-TNF*	
*Gene Name*	log2 *Fold-change* LFQ	log2 *Fold-change* SILAC	log2 *Fold-change* LFQ	log2 *Fold-change* SILAC	log2 *Fold-change* LFQ	log2 *Fold-change* SILAC
**Akr1b1**	*0*	*0*	*0.4508*	0.5497	*0.5960*	0.6343
**Akr1b3**	*0*	*ND*	0.4508	*ND*	0.5960	*ND*
**Akr1b7**	*0*	*0*	1.2905	*1.7506*	1.4273	*ND*
**Akr1b8**	*0*	*0*	1.4440	1.7608	1.5898	2.1034
**Blvrb**	−0.3809	−0.2301	*0*	0.5764	0.7862	0.4835
**Cat**	*0*	*0*	1.0800	1.1262	1.0148	1.2222
**G6pdx**	*0*	*0*	0.5651	0.5211	0.7523	0.5877
**Gsta3**	*0*	*ND*	1.2664	*ND*	0.9136	*ND*
**Gstp1**	−0.3949	−0.1888	0.9105	1.5843	1.2616	1.7841
**Hmox1**	−0.2424	−0.1233	0.8703	0.7048	0.6607	0.4045
**Nfkb2**	*1.1143*	1.3995	*0*	*ND*	*0*	−0.2200
**Nqo1**	*0*	*ND*	1.7907	*ND*	1.6534	*ND*
**Pgd**	*0*	0.1012	*0.5913*	0.4975	*0.6452*	0.5666
**Por**	*−0.0862*	*ND*	*0.0429*	*ND*	0.3405	0.1561
**Prdx1**	*0*	*0*	0.2915	*0.2540*	0.4062	*0.2665*
**Prdx5**	*0*	*0.1132*	0.4444	*0.5167*	0.6037	*0.3645*
**Ptgr1**	*0*	*0*	*0.9705*	1.2600	*0.9978*	1.2999
**Sqstm1**	−0.3753	−0.6061	0.4559	0.8246	0.4579	1.3186
**Taldo1**	*0*	*ND*	0.5603	0.7171	0.9184	0.8525
** Tkt	*−0.1891*	*0*	0.4719	0.5824	0.4815	0.5555
**Txn1**	−0.5145	*0*	*0*	*0.2655*	0.4939	*0.2589*
**Sod2**	1.6735	1.8043	0.7816	0.5633	0.6954	0.1391

** *p* < 0.005.

## Data Availability

The data presented in this study are available on request from the corresponding author. The data are not publicly available.

## Supplementary Materials

The following supporting information can be downloaded at: https://www.mdpi.com/article/10.3390/antiox11081577/s1, Table S1: Compounds identified or putatively identified by LC-HRMS in negative and positive ion modes with the targeted method ordered on the basis of the retention time; Table S2: Compounds putatively identified by LC-HRMS in negative and positive ion modes with the untargeted method ordered on the basis of the retention time.

## Abbreviations

LC-HRMS	Liquid chromatography–high-resolution mass spectrometry
ESI	Electrospray ionization
NRF2	Nuclear factor erythroid 2-related factor 2
NF-κB	Nuclear factor kappa-light-chain-enhancer of activated B cells
SILAC	Stable isotope labeling by amino acids in cell culture
TAP	Thinned apple polyphenols
TNF-α	Tumor necrosis factor
IL1-α	Interleukin 1 alpha
GSH	Glutathione
NADPH	Reduced nicotinamide adenine dinucleotide phosphate
CVD	Cardiovascular diseases
LDL	Low-density lipoprotein
ICAM-1	Intercellular adhesion molecule 1
CRP	C-reactive protein
IL-6	Interleukin 6
LBP	Lipopolysaccharide-binding protein
KEAP1	Kelch-like ECH-associated protein 1
HMOX1	Heme oxygenase 1
MMPs	Matrix metalloproteinases
MAPK	Mitogen-activated protein kinases
TLC	Thin-layer chromatography
CAT	Catalase
AKR	Aldo-keto reductases
NQO1	NAD(P)H dehydrogenase quinone 1
SQSTM1	Sequestosome 1
TKT	Transketolase
PGD	Phosphogluconate dehydrogenase
G6PDX	Glucose-6-phosphate 1-dehydrogenase X
TALDO1	Transaldolase 1
PTGR1	Prostaglandin reductase 1
BLVRB	Biliverdin reductase B
SOD2	Superoxide dismutase 2
AKT	Serine/threonine kinase 1
IKKB	Inhibitor of nuclear factor kappa-B kinase subunit beta
GSTA3	Glutathione S-transferase alpha 3
GSTP1	Glutathione S-transferase Pi 1
POR	Cytochrome P450 oxidoreductase
PRDXs	Peroxiredoxins
TXN1	Thioredoxin 1
GXP	Glutathione peroxidases
ME-1	Malic enzyme-1
IDH1	Isocitrate dehydrogenase-1
CO	Carbon monoxide
BACH1	BTB domain and CNC homolog 1
AP-1	Activator protein 1
