# Supplementary material for: Polyphenols from Thinned Young Apples: HPLC-HRMS Profile and Evaluation of Their Anti-Oxidant and Anti-Inflammatory Activities by Proteomic Studies
Source: Antioxidants (Basel). 2022 Aug 15;11(8):1577. doi: 10.3390/antiox11081577 (PMC9405250; doi:10.3390/antiox11081577)
Supplement: Supplementary file 1 [file antioxidants-11-01577-s001.zip › antioxidants-1826844-supplementary.pdf]

Table S1. Compounds identified or putatively identified by LC -HRMS in negative and positive ion modes with the targeted method ordered on the basis of the retention time.

Peak	Compound name	RT	$[M-H]^{-}_{exp}$	Δ ppm	MS/MS	$[M+H]^{+}_{exp}$	Δ ppm	MS/MS	Identification strategy
	Organic and phenolic acids								
1	Citric acid	2.3	191.01979	0.606	173	-			[34]
2	Protocatechuic acid glucoside	2.7	315.07196	0.286	153-109	-			[33]
3	Caffeoylquinic acid isomer 1	3.5	353.08673	0.006	191-179-135	-			[28-29,31,33-36,44]
3	Protocatechuic acid	3.7	153.01971	0.962	109	-			Standard
3	Glutathionyl chlorogenic acid	3.8	658.15118	-0.641	529-466-385-272-193-191	660.16360	-3.211	642-585-531-264	[32]
3	Caffeoyl glucoside isomer 1	3.8	341.08752	0.238	251-179	-			[35]
3	Caffeoyl glucoside isomer 2	4.3	341.08716	0.133	179-251-281	343.10120	-3.323	-	[35]
4	Coumaroylquinic acid isomer 1	5.2	337.09229	0.147	191-163-119	339.10730	-0.383	147	[31,38]
4	Caffeoyl glucoside isomer 3	5.2	341.08660	-0.032	281-251-179	343.10110	-3.614	-	[35]
4	Caffeoylquinic acid isomer 2	5.7	353.08649	-0.062	191-179-135	355.10245	-1.239	163	[28-29,31,33-36,44]
5	*p*-Coumaric acid-O-glucoside isomer 1	6.0	325.09265	0.264	163-119	-			[31]
5	*p*-Coumaric acid-O-glucoside isomer 2	6.9	325.09265	0.264	163-145-119	327.10760	1.253	-	[31]
6	Caffeic acid	7.6	179.03577	1.052	135	181.04961	-2.541	163	Standard
6	Ferulic acid-O-glucoside isomer 1	7.8	355.10263	0.077	235-193	357.11800	2.324	-	[31]
7	*p*-Coumaric acid-O-glucoside isomer 3	8.3	325.09216	0.113	163-119	327.10750	0.948	309-165-147	[31]
8	Ferulic acid-O-glucoside isomer 2	9.1	355.10278	0.119	295-265-235-193	357.11790	2.044	339-177	[31]
8	Coumaroylquinic acid isomer 2	9.4	337.09229	0.147	173-163	339.10720	-0.678	147	[31,38]
9	Ferulic acid-O-glucoside isomer 3	10.8	355.10284	0.136	295-265-235-193	357.11810	2.604	339-177	[31]

12	*p*-Coumaric Acid	13.1	163.04041	0.882	119	165.05453	-3.756	147	Standard
28	Dicaffeoylquinic acid	29.3	515.11853	0.025	353-191-179	517.13320	0.251	-	[31,38]
	Flavanols								
4	Catechin	5.4	289.07153	0.299	245-205-271-179 5	291.08636	-1.649	273-165-151-139-123	Standard
5	Procyanidin B2	6.9	577.13354	-0.088	559-451-425-407-289	579.14850	-2.987	427-409-291	[29,34-35,42-43]
7	Epicatechin	8.7	289.07144	0.268	245-205-179	291.08643	-1.409	273-165-151-139-123	Standard
10	Procyanidin trimer	11.0	865.19183	-0.648	695-577-449	867.21295	-0.773	715-577-289	[28,34-35,41]
12	Procyanidin tetramer	12.9	1153.25208	-0.758	1135-739-577	1155.27502	-1.723	-	[41]
13	Procyanidin trimer	13.8	865.19556	-0.217	695-577-407				[28,34-35,41]
14	Procyanidin pentamer	15.1	-			1443.33911	-0.887	-	[41]
14	Procyanidin hexamer	15.1	-			866.20331 $[M+2H]^{2+}$	-4.237	-	[37]
17	Procyanidin heptamer	19.0	-			1010.23633 $[M+2H]^{2+}$	-2.323	-	[37]
19	Procyanidin octamer	20.4	-			1154.26868 $[M+2H]^{2+}$	-1.455	-	[37]
21	Procyanidin nonamer	21.5	-			1298.29871 $[M+2H]^{2+}$	-2.582	-	[37]
	Flavanones								
15	Naringenin glucoside (prunin)	17.0	433.11041	-0.580	271	435.12848	-1.425	-	Standard
18	Eriodictyol-hexoside	20.1	449.10785	0.003	287	451.12317	-1.884	289	[34]
30	Naringin (Naringenin 7-O-neohesperidoside)	30.7	-			581.18567	-2.306	435-273	[31,36]
34	Eriodictyol	42.9	287.05579	0.270	151-135	289.07101	-0.657	-	[31,36]
38	Naringenin	53.1	271.06076	0.243	177-151	273.07584	-1.611	-	[31,36,39]
	Flavonols								
16	Kaempferol-glucoside	18.9	447.09161	-0.129	285	449.10751	-1.915	287	[29,31,36]
20	Quercetin-3-O-glucoside	21.0	463.08673	-0.080	301	465.10236	-1.978	303	[28,30-31,34-35,40-41,44]
22	Quercetin-3-O-galactoside	22.5	463.08694	-0.035	301	465.10211	-2.516	303	Standard

24	Quercetin pentoside isomer 1	24.8	433.07687	0.079	301	435.09180	-2.114	303	[28,30-31,34-35,40-41,44]
26	Quercetin pentoside isomer 2	28.0	433.07675	0.051	301	435.09171	-2.321	303	[28,30-31,34-35,40-41,44]
28	Quercetin pentoside isomer 3	29.0	433.07715	0.143	301	435.09186	-1.977	303	[28,30-31,34-35,40-41,44]
29	Quercetin-3-O-rhamnoside	29.8	447.09262	0.097	301	449.10730	-2.382	303	Standard
35	Quercetin	48.0	301.03476	0.159	273-232-179-151	303.04999	-1.551	285-257-229-165-153-137	Standard
40	Kaempferol	54.7	285.04013	0.268	155	287.05511	-1.533	-	Standard
40	Isorhamnetin	54.7	315.05054	0.194	300	317.06567	-1.388	302	[36]
	Dihydrochalcones								
27	Phloretin-O-xyloglucoside isomer 1	28.8	567.17010	-0.128	273-167	569.18561	-2.460	437-419-275	[28,31,33-35,41-42]
29	Phloretin-O-xyloglucoside isomer 2	30.0	567.17072	-0.019	273-167	569.18579	-2.143	437-275	[28,31,33-35,41-42]
31	Phlorizin	35.0	435.12845	-0.152	273	437.14401	-1.693	275	Standard
39	Phloretin	54.1	273.07593	0.066	167	275.09190	-0.109	169-149-127-107	Standard
	Flavones								
36	Luteolin	49.1	285.04013	0.268	241-217-199-175-151	287.05527	-0.975	269-245-153	Standard
	Triterpenoids								
44	Euscaphic acid	65.0	487.34171	-0.127	-	489.35681	-2.370	471-453-443-425-407	[41]

Table S2. Compounds putatively identified by LC -HRMS in negative and positive ion modes with the untargeted method ordered on the basis of the retention time.

Peak	Compound name	RT	$[M-H]^{-}_{exp}$	Δ ppm	MS/MS	$[M+H]^{+}_{exp}$	Δ ppm	MS/MS	Identification strategy
	Organic and phenolic acids								
11	3-(benzoyloxy)-2-hydroxypropyl glucopyranosiduronic acid	11.8	371.09741	0.037	249-121	373.11310	0.509	-	[52]
13	Sinapoyl hexoside	13.9	385.11290	-0.006	223-205				[52]
37	3-[2,4,5-trihydroxy-3-(3-methylbut-2-en-1-yl) phenyl] propanoic acid	51.8	265.10748	0.163	247-221-203-177-151-87	267.12290	0.824	249-221	[27]
	Dihydrochalchones								
25	Hydroxyphloretin glucoside	26.2	451.12428	0.176	433-313-289	453.13900	-0.265	435-291-235-165	FooDB
	Flavonols								
20	Patuletin hexoside isomer 1	20.8	493.09738	-0.058	331-316	495.11260	-1.414	333	PubChem
22	Patuletin hexoside isomer 2	22.5	493.09698	-0.139	331-316	495.11270	-1.212	333	PubChem
23	Patuletin hexoside isomer 3	23.8	493.09747	-0.040	331-316	495.11240	-1.818	333	PubChem
27	Patuletin pentoside	28.4	463.08682	-0.179	331-316	465.10200	-1.591	333	Patuletin fragment and loss of a pentoside (-132 Da)
29	Patuletin rhamnoside	30.0	477.10352	0.161	316-331	479.11740	-2.066	333	Patuletin fragment and loss of a rhamnoside (-146 Da)
32	Isorhamnetin rhamnoside	38.5	461.10745	-0.084	315-300	463.12300	-1.015	317	Isorhamnetin fragment and loss of a rhamnoside (-146 Da)
33	Kaempferol rhamnoside	39.1	431.09695	-0.075	285	433.11280	-0.254	287	Kaempferol fragment and loss of a rhamnoside (-146 Da)
36	Methoxyquercetin (patuletin)	49.3	331.04520	0.107	316-285-209-181	333.06110	1.862	318	HMDB
37	Quercetin 3-(3-*p*-coumaroylglucoside)	52.1	609.12440	0.085	301	611.13890	-0.998	303	HMDB
	Flavanones								
38	Eriodictyol 7-(6-trans-*p*-coumaroylglucoside)	53.5	595.14441	-0.034	287-307-459-449	597.15940	-1.423	579-331-289	CFM-ID
	Lipids								

42	(10E,15Z)-9,12,13-Trihydroxy-10,15-octadecadienoic acid	55.7	327.21707	0.146	291-229-211-171	351.21414 [M+Na]$^+$	-1.680	333-315-235	mzCloud
43	Trihydroxy-octadecenoic acid	57.2	329.23251	0.082	293-229-211-183-171	353.22964 [M+Na]$^+$	-2.095	335-317-235	mzCloud
